# Role of Atrial Fibrillation Threshold Evaluation on Guiding Treatment

**Published:** 2003-10-01

**Authors:** Takeshi Shirayama

**Affiliations:** Kyoto Prefectural University of Medicine, Department of Medicine, Division of Cardiology, Kawaramachi Hirokoji, Kamigyo-ku, Kyoto, 602-8566, Japan

**Keywords:** atrial fibrillation, electrophysiological test, therapy, prognosis

## Abstract

Atrial fibrillation could be induced reproducibly by 50Hz rapid stimulation which was given through systolic and early diastolic phase of atrial excitation. Duration of atrial fibrillation induced in this way was roughly dependent on the current amplitude of the stimulation. The minimum current that could induce long-lasting atrial fibrillation (30sec in the clinical setting, 2sec in the rabbit or rat model) was defined as atrial fibrillation threshold (AFT). AFT was larger in patients who had history of atrial fibrillation than those who did not. Anti-arrhythmic drugs raised AFT by various degrees both in experimental and clinical cases. Long-term efficacy of a drug could be predicted in a patient, measuring how much the drug increased AFT (cut-off point = 5mA increase). AFT is a useful marker to evaluate atrial vulnerability and to guide pharmacological treatment of atrial fibrillation.

## Introduction

Atrial fibrillation is a target of intensive research because fibrillation is the final frontier in the arrhythmology [1-3]. Now we have several therapeutic modalities for this common arrhythmia, including pharmacological and non-pharmacological approach. However, long-term success rate of these treatments would be 50% to 60 % at the best [4] . There are several reasons for its difficulty: 1)this arrhythmia is essentially due to aging in clinical cases, 2)the mechanism of this arrhythmia is not fully understood [5] , 3)there is no appropriate animal model [6] . We have been working on these problems for several years, and become to use atrial fibrillation threshold as a tool to evaluate the easiness to induce atrial fibrillation (atrial vulnerability) both in the experimental [7-10] and the clinical [11-13] settings. This is the unique parameter to evaluate atrial vulnerability quantitatively [12,13] . Because very few researches have been done in this field, I will summarize the data from our laboratory, comparing with the ventricular counterpart in this review article.

## What is fibrillation threshold ?

**Figure 1: Strength-interval relations in provoking fibrillation:**
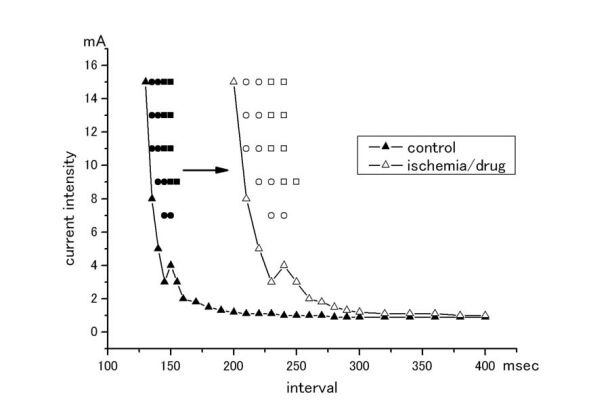
Figure shows an example of strength-interval relations. Triangles are pacing threshold at each coupling interval after an initial deflection of atrial/ventricular activity. Circles indicate induction of repetitive responses, and squares show induction of fibrillation. Closed symbols are control data and open symbols are data obtained in the presence of ischemia or procainamide.

Fibrillation threshold has been studied mostly in the ventricle [14-16] since Wiggers et al [17,18]. A single stimulus was given to induce ventricular fibrillation in the diseased and the normal hearts. For this purpose, the stimulus should be given at a critical time point with sufficient current amplitude. The time point is usually located at around the peak of T wave of ECG. The minimum current amplitude to induce fibrillation is defined as fibrillation threshold. This concept is based on a fact that fibrillation can be induced by larger current than a critical (threshold) amplitude. Recently, it is recognized that the second critical amplitude exists when current amplitude is increased incrementally. Larger current than the second critical point can not induce fibrillation, but rather terminate fibrillation [19,20]. This point is called defibrillation threshold, which is now widely studied because of implantable cardioverter defibrillators in clinical use. In the combination with vulnerable period (the zone of the critical time points to induce fibrillation), there is an area in the strength-interval curve where fibrillation can be induced by a single stimulus [17,18]. This area is shifted rightwards along the axis of coupling interval, for example, when myocardial ischemia or anti-arrhythmic drugs are introduced [21] (Figure 1). The same argument could be applied to the atrium. However, atrial vulnerable period was less defined than that of the ventricle [22,23]. Atrial T wave is barely discernable in a standard ECG.

**Figure 2: Measurement of atrial fibrillation threshold:**
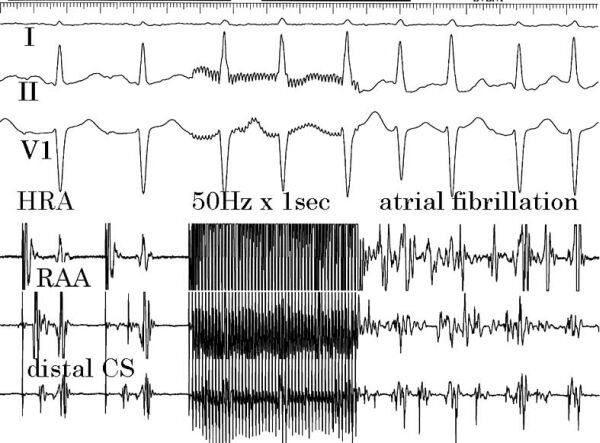
Three leads of a standard ECG record and 3 intra-cardiac records are shown. HRA, RAA and CS are high right atrium, right atrial appendage and coronary sinus, respectively. In the middle of the figure, a rapid pacing at 50Hz was applied to HRA for 1sec, which was induced atrial fibrillation. A constant pacing at a rate of 120/min preceded a rapid pacing. Duration of atrial fibrillation was measured, and then current amplitude of the rapid pacing was increased for the next trial. The minimum current that could induce “sustained” fibrillation was defined as atrial fibrillation threshold.

There have been several methods to induce ventricular fibrillation [14,17]. Among them, a continuous high frequency pacing at 50-100Hz which covers a whole “vulnerable period” can induce fibrillation consistently [14]. Thus we applied this type of stimulation to the atrium and the minimum current to induce long-lasting atrial fibrillation (>30sec in clinical settings, >2sec in experiments) was defined as fibrillation threshold (Figure 2). Duration of “long-lasting” atrial fibrillation was selected rather arbitrarily as the definition but it was based on the relationship between the current intensity and the duration (see Matsuo et al [9]). The duration was positively correlated with the current intensity until the current reached the threshold. This definition is different from conventional usage in terms of the stimulating pattern.

## Mechanism of induction of fibrillation

The precise mechanism of induction of fibrillation by a single pulse or train pulses is not clear. However, there are some observational studies in the ventricle [14,15,17,19]: 1)localized repetitive activities were induced at the stimulating site, 2)excitations were propagated, 3)repetitive excitations were observed at many sites of the myocardium 4)excitations resulted in disorganized activity (fibrillation) because of inhomogeneity of conduction and repolarization. When high frequency stimulation is given to the ventricle at an intensity of diastolic threshold, ventricular response mimicking ventricular tachycardia could be observed. As the intensity is raised incrementally, the intervals of ventricular excitation become shorter. At and beyond a critical intensity of the stimulating current, ventricular excitations become fibrillation. Although these results are based on the ventricular study, it is probable that the same phenomenon could be observed in the atrium.

## Experimental methodology of induction of atrial fibrillation

Most popular method to induce atrial fibrillation would be an extra-stimulus method, that is, an extra-stimulus is given to the atrium at a critical time point after basic conditioning stimulations at a fixed rate [23]. Because reproducibility of this method is relatively low, statistical evaluation is often necessary after many trials [24] to evaluate drug efficacy or atrial vulnerability. On the other hand, continuous rapid pacing could easily induce fibrillation. Thus this method was introduced for the quantitative evaluation of atrial vulnerability (easiness to induce atrial fibrillation). We chose 50Hz for 1sec stimulation for this purpose. The increment of the current intensity is 1mA for the clinical setting, and 0.1 to 0.5mA for the experimental use.

## Experimental results

Atrial fibrillation threshold has been measured in the isolated hearts of guinea pig or rabbit. Sodium channel blockers [7,8,11] (disopyramide, pilsicainide, flecainide, aprindine, lidocaine), potassium channel blockers [7,10] (E-4031), amiodarone, and SD-32129 raised atrial fibrillation threshold by various degrees. Although the increase of atrial fibrillation threshold induced by anti-arrhythmic drugs was positively related to the increase of effective refractory period, E-4031 had the least potency [7]. This results could be explained by the fact that sodium channel blockers made refractory period longer than E-4031 did because of longer post-excitation refractoriness after a high frequency stimulation [25]. Note that the blocking effect of potassium channels by Ikr blockers (such as E-4031) is less prominent when the excitation frequency becomes higher (reverse use-dependent block [26], but see Ohler et al [27]). In the presence of anti-arrhythmic drugs in the perfusing solution, it was more difficult to induce repetitive atrial firing or atrial fibrillation by electrical stimulation. At the same time, atrial fibrillation threshold was increased.

Effective refractory period becomes shorter when a rapid pacing is continuously applied to the atrium. This phenomenon is called “atrial electrical remodeling” [28], which is accompanied by the reduction of L-type calcium current and transient outward current [2,29]. Expression of novel channels was also reported [30]. Large numbers of experimental studies have been performed regarding atrial remodeling. In these studies, single extra-stimulus or burst pacing similar to ours has been used to induce atrial fibrillation. However, atrial vulnerability was compared in terms of the duration of atrial fibrillation that was induced, but not the intensity of the stimulation. These studies did not evaluate how easy to induce atrial fibrillation, but how difficult to recover from the arrhythmia. The evaluation of the duration of the arrhythmia disclose its stability after the physiological and the biochemical changes of the atrium were implemented, but it could not be suitable to evaluate the propensity of the patients to paroxysmal attack of atrial fibrillation. In our experience, a stronger current to the atrium could induce longer atrial fibrillation until the current reached the fibrillation threshold [12].

## Clinical feasibility

Experimental data suggested that the measurement of the threshold could predict the easiness to induce atrial fibrillation. Indeed atrial fibrillation threshold was lower in the patients who had a history of atrial fibrillation (median 11mA) than normal control (median 5mA) [12] . When cut-off point was set at 10mA, the sensitivity and the specificity were 94%, and 60%, respectively. Effective refractory period, conduction time, or other indicators of atrial vulnerability were not different between two groups. The secondary indicators to distinguish the patients with atrial fibrillation from normal subjects were %maximum atrial fragmentation (%MAF) and fragmented activity zone (FAZ) [31]. Their sensitivity and specificity were 78%, 52% (%MAF), and 47%, 84% (FAZ), respectively. %MAF is the relative increase of the width of local atrial electrogram by the extra-stimulation. FAZ is the zone of coupling intervals that made local atrial electrogram longer by 50% or more.

Atrial fibrillation threshold (AFT) could be affected by the autonomic tone because isoproterenol infusion decreased AFT (unpublished data). The data obtained on the day when the catheters were inserted were lower than those obtained on the following days. Thus the results of the measurement should be interpreted cautiously. However, reproducibility of AFT was excellent (±1mA, r=0.95 on the regression line) during a short term examination (<3 hours), or if it was measured on the next day or later after the catheters were inserted.

## Evaluation of drug efficacy in the clinical setting

The benefit of the AFT measurement would be quantitative evaluation of the drug efficacy. Anti-arrhythmic drugs raised AFT in general, but the increase of AFT by a drug was different in the individual patient. For example, procainamide raised AFT by 5mA in a patient, but propafenone did not change AFT at all in the same patient. In this way, we could categorize anti-arrhythmic drugs into 2 groups in the individual patient, i.e. drugs that raise AFT significantly, and drugs that does not increase AFT in the patient. The effective drugs in a patient were not necessarily the same in another patient. When the cut-off point of effectiveness was set to 5mA increase, “effective” drug could prevent 88% of the patients from the recurrence of atrial fibrillation at least for 1 year whereas only 13% were free from atrial fibrillation with “ineffective” drugs [13]. Thus this method could be used to select effective drugs in an individual patient.

However, the prediction of the effectiveness did not mean the high efficacy of the drug in a large population. As previously reported[1,4], efficacy of each drug against atrial fibrillation was around 40 to 50% in our study. AFT is a method to select beneficial drugs to the specific patient.

 Serial electrophysiological test has been less recommended in case of ventricular tachycardia, because “effective drug” determined in terms of inducibility did not improve the prognosis of the patients [32]. On the other hand, AFT does not depend on all-or-none type determination of inducibility, but it determines how strong is the drug effect against the arrhythmia. As a matter of fact, atrial fibrillation was always induced in this method because of the definition of AFT.

## Rate control and rhythm control

Clearly, AFT measurement determines a drug suitable to maintain sinus rhythm. Recently, the results of the Atrial Fibrillation Follow-up Investigation of Rhythm Management (AFFIRM) study were published [33]. This study did not show the advantage of maintaining sinus rhythm over heart rate control in terms of prognosis. This result may argue against the usefulness of AFT measurement. However, mortality of the patients who suffered from paroxysmal atrial fibrillation is relatively low. The biggest concern of the patients is often its symptom. Thus, when mortality is not different, it would be desirable to select less symptomatic life. In this sense, pharmacological treatment with class I anti-arrhythmic drugs could still be a first line therapy for “lone” atrial fibrillation.

## AFT in the future

AFT is a quantitative marker to select effective drugs against atrial fibrillation. The concept of this method is “custom-made” medicine. Large-scale population study would reveal overall efficacy of the drug, but a negative side is negligence of individuality or specificity of each patient. Because the efficacy of pharmacological and non-pharmacological treatment is still not high, it is important to know which method should be applied first. It is possible to measure AFT first, and if “effective” drug could be found, the patient should be treated with the regimen. If “effective” drug is not found, catheter ablation or some other non-pharmacological approach should be considered.

## References

[R1] Khairy P, Nattel S (2002). New insights into the mechanisms and management of atrial fibrillation. CMAJ.

[R2] Bosch RF, Nattel S (2002). Cellular electrophysiology of atrial fibrillation. Cardiovasc Res.

[R3]  Nattel S, Li D, Yue L (2000). Basic mechanisms of atrial fibrillation--very new insights into very old ideas. Annu Rev Physiol.

[R4] Wijffels MC, Crijns HJ (2002). Non-invasive characteristics of atrial fibrillation: the value of Holter recordings for the treatment of AF. Card Electrophysiol Rev.

[R5] Brundel BJ, Henning RH, Kampinga HH (2002). Molecular mechanisms of remodeling in human atrial fibrillation. Cardiovasc Res.

[R6] Gaspo R (1999). The tachycardia-induced dog model of atrial fibrillation. Clinical relevance and comparison with other models. Pharmacol Toxicol Methods.

[R7] Inoue M, Inoue D, Ishibashi K (1994). Effects of E-4031 on atrial fibrillation threshold in guinea pig atria: comparative study with class I antiarrhythmic drugs. J Cardiovasc Pharmacol.

[R8] Inoue M, Inoue D, Ishibashi K (1993). Effects of pilsicainide on the atrial fibrillation threshold in guinea pig atria. A comparative study with disopyramide, lidocaine and flecainide. Jpn Heart J.

[R9] Matsuo R, Shirayama T, Inoue K (1999). SD3212, a new antiarrhythmic drug, raises atrial fibrillation threshold in isolated rabbit hearts. Heart Vessels.

[R10] Shirayama T, Sakamoto T, Yamamura M, Tse H-F, Lee KL, Lau CP (2003). Mechanism of shortening of atrial refractory period after short term rapid pacing. Clinical Cardiac Pacing and Electrophysiology.

[R11] Ishibashi K, Inoue D, Sakai R (1995). Effects of disopyramide on the atrial fibrillation threshold in the human atrium. Int J Cardiol.

[R12] Inoue K, Shirayama T, Shiraishi H (2001). Clinical significance of the atrial fibrillation threshold in patients with paroxysmal atrial fibrillation. Pacing Clin Electrophysiol.

[R13] Shirayama T, Shiraishi H, Yoshida S (2002). Atrial fibrillation threshold predicted long-term efficacy of pharmacological treatment of patients without structural heart disease. Europace.

[R14] Sugimoto T, Schaal SF, Wallace AG (1967). Factors determining vulnerability to ventricular fibrillation induced by 60cps alternating current. Circ Res.

[R15] Toda I, Murakami Y, Nozaki A (1988). Ventricular fibrillation threshold measured by continuous 50cps stimulation for the evaluation of the antifibrillatory effect of the drugs. Jpn Circ J.

[R16] Kostis JB, Goodking MJ, Gotzoyannis S (1977). Effect of lidocaine on the atrial fibrillation threshold. Am Heart J.

[R17] Wiggers CJ, Wegria NF (1940). Ventricular fibrillation due to single, localized induction and condensor shocks applied during the vulnerable phase of ventricular systole. Am J Physiol.

[R18] Wiggers CJ, Wegria R (1940). Quantitative measurement of fibrillation threshold of mammalian ventricles with observations on the effect of procaine. Am J Physiol.

[R19]  Karagueuzian HS, Chen PS (2001). Cellular mechanism of reentry induced by a strong electrical stimulus: implications for fibrillation and defibrillation. Cardiovasc Res.

[R20] Chen PS, Feld GK, Kriett JM (1993). Relation between upper limit of vulnerability and defibrillation threshold in humans. Circulation.

[R21] Michelson EL, Spear JF, Moore Neil E (1981). Effects of procainamide on strength-interval relations in normal and chronically infarcted canine myocardium. Am J Cardiol.

[R22] Orias O, Gilbert JL, Siebens AA (1950). Effectiveness of single rectangular electrical pulses of known duration and strength in evoking auricular fibrillation. Am J Physiol.

[R23] Brooks McC C, Orias O, Gilbert JL (1951). Auricular fibrillation: relationship of ‘vulnerable period’ to ‘dip’ phenomenon of auricular excitability curve. Am J Physiol.

[R24] Shinagawa K, Shiroshita-Takeshita A, Schram G (2003). Effects of antiarrhythmic drugs on fibrillation in the remodeled atrium: insights into the mechanism of the superior efficacy of amiodarone. Circulation.

[R25] Kanki H, Mitamura H, Takatsuki S (1998). Postrepolarization refractoriness as a potential anti-atrial fibrillation mechanism of pilsicainide, a pure sodium channel blocker with slow recovery kinetics. Cardiovasc Drugs Ther.

[R26] Wettwer E, Scholtysik G, Schaad A (1991). Effects of the new class III antiarrhythmic drug E-4031 on myocardial contractility and electrophysiological parameters. J Cardiovasc Pharmacol.

[R27] Ohler A, Amos GJ, Wettwer E (1994). Frequency-dependent effects of E-4031, almokalant, dofetilide and tedisamil on action potential duration: no evidence for "reverse use dependent" block. Naunyn Schmiedebergs Arch Pharmacol.

[R28] Wijffels MCEF, Kirchhof CJHJ, Dorland R (1995). Atrial fibrillation begets atrial fibrillation: a study in awake chronically instrumented goats. Circulation.

[R29] Allessie M, Ausma J, Schotten U (2002). Electrical, contractile and structural remodeling during atrial fibrillation. Cardiovasc Res.

[R30] Yamashita T, Murakawa Y, Hayami N (2000). Short-term effects of rapid pacing on mRNA level of voltage-dependent K(+) channels in rat atrium: electrical remodeling in paroxysmal atrial tachycardia. Circulation.

[R31] Ohe T, Matsuhisa M, Kamakura S (1983). Relation between the widening of the fragmented atrial activity zone and atrial fibrillation. Am J Cardiol.

[R32] Mason JW (1993). A comparison of seven antiarrhythmic drugs in patients with ventricular tachyarrhythmias. Electrophysiologic Study versus Electrocardiographic Monitoring Investigators. N Engl J Med.

[R33] Wyse DG, Waldo AL, DiMarco JP (2002). A comparison of rate control and rhythm control in patients with atrial fibrillation. N Engl J Med.

